# AI-empowered integrative structural characterization of m^6^A methyltransferase complex

**DOI:** 10.1038/s41422-022-00741-8

**Published:** 2022-11-10

**Authors:** Xuhui Yan, Kai Pei, Zeyuan Guan, Feiqing Liu, Junjun Yan, Xiaohuan Jin, Qiang Wang, Mengjun Hou, Chun Tang, Ping Yin

**Affiliations:** 1 National Key Laboratory of Crop Genetic Improvement, Hubei Hongshan Laboratory, Huazhong Agricultural University, Wuhan, Hubei China; 2 Beijing National Laboratory for Molecular Sciences, College of Chemistry and Molecular Engineering, PKU-Tsinghua Center for Life Sciences, Center for Quantitative Biology, Peking University, Beijing, China

**Keywords:** Cryoelectron microscopy, RNA modification

Dear Editor,

*N*^6^-methyladenosine (m^6^A) is the most abundant and prevalent internal modification in mRNA.^1^ In mammals, m^6^A exerts pivotal roles in posttranscriptional regulation and its dysregulation is implicated in various diseases including cancer.^2^ m^6^A is installed by a multicomponent methyltransferase complex (MTC, also known as the m^6^A writer complex).^3,4^ The mammalian MTC is composed of the core m^6^A methyltransferase METTL3–METTL14 complex (MTC core) and several regulatory proteins including WTAP, the adaptor responsible for METTL3–METTL14 localization and proper substrate recruitment,^5^ and VIRMA (KIAA1429), the specificity mediator that mediates preferential m^6^A modification at the 3′ untranslated regions (UTRs, Fig. 1a).^6^ Dysregulation of MTC components results in the disruptions of m^6^A.^2^ Compared to the other currently identified regulators (HAKAI, ZC3H13 and RBM15), WTAP and VIRMA are reported to have greater impacts on total mRNA m^6^A levels upon knockdown.^5,6^ Despite the advances in understanding the roles of individual MTC components and the structural determination of MTC core,^7–9^ the overall molecular architecture of the m^6^A writer holocomplex is missing. Here, we report the cryogenic electron microscopy (cryo-EM) structure of human WTAP–VIRMA (3.1 Å) in the METTL3–METTL14–WTAP–VIRMA (M–M–W–V) complex and modeled a structure of the quaternary M–M–W–V complex based on AlphaFold2 predictions and structural restraints from intermolecular chemical crosslinking mass spectrometry (CXMS).

**Fig. 1:**
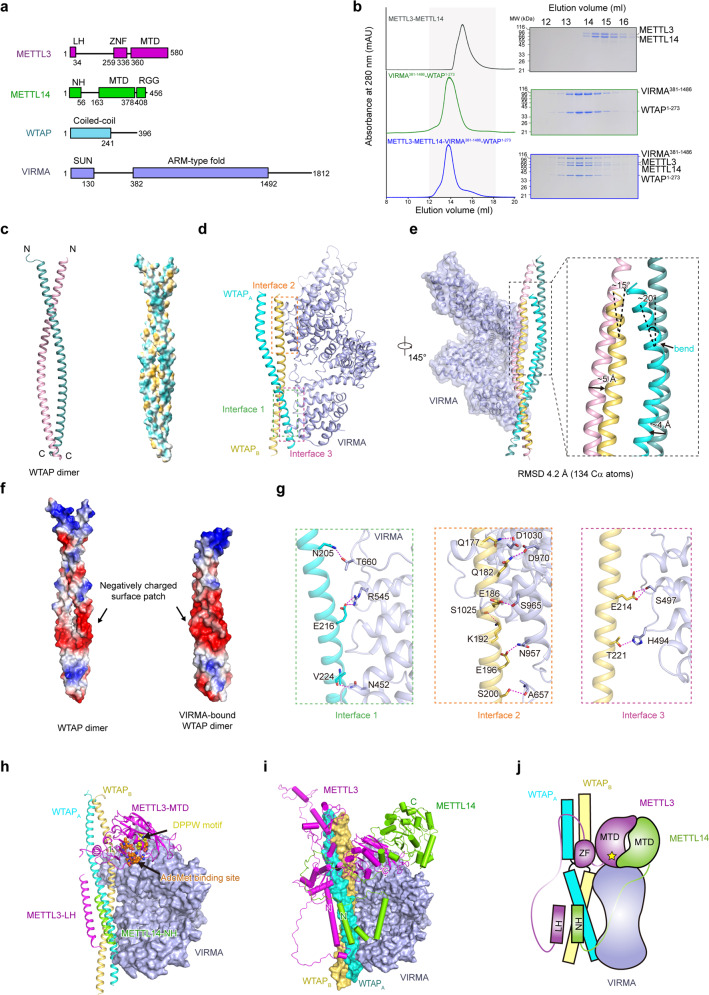
Structure of the human m^6^A writer complex. **a** Schematic diagram of the domain information of METTL3, METTL14, WTAP and VIRMA. LH leader helix, ZFD zinc finger domain, MTD MTase domain, NH N-terminus helix. **b** Gel filtration analysis of the human METTL3–METTL14–WTAP^1–273^–VIRMA^381–1486^ complex. **c** The crystal structure of WTAP alone. Two molecules of WTAP form a symmetric parallel α-helical coiled-coil, with the two chains colored light pink and light teal, respectively. **d** The cryo-EM structure of WTAP–VIRMA complex in the METTL3–METTL14–WTAP^1–273^–VIRMA^381–1486^ quaternary complex. Three interaction interfaces can be observed between WTAPs and VIRMA. WTAP_A_, cyan; WTAP_B_, orange. **e** Comparison of the crystal structure of WTAP alone and the cryo-EM structure of VIRMA-bound WTAPs. VIRMA-bound WTAPs bend around the observed N-termini. **f** Electrostatic surfaces of WTAP alone (PDB: 7YFJ) and VIRMA-bound WTAPs (PDB: 7YG4). **g** Details of the observed interaction interfaces between WTAP and VIRMA. **h**, **i** Structural model of the METTL3–METTL14^1–399^–WTAP^148–237^–VIRMA^342–1292^ complex based on CXMS measurement and AI prediction. METTL3 and METTL14 are shown in green and magenta, respectively. METTL3 LH is docked close to WTAP, and the MTC core catalytic center is docked close to VIRMA, with the DPPW motif and AdoMet binding site positioned towards a cleft between WTAP and VIRMA. **j** Schematic diagram of the modeled METTL3–METTL14^1–399^–WTAP^148–237^–VIRMA^342–1292^ complex.

Utilizing a baculovirus-insect cell expression system, we obtained the METTL3–METTL14 complex (Fig. 1b) and investigated the interaction between METTL3–METTL14, WTAP, and VIRMA through co-expression and purification. WTAP was observed to form a complex with the MTC core, while VIRMA can only form a stable complex with the MTC core in the presence of WTAP (Supplementary information, Fig. S1). The methyl transfer activity of the quaternary M–M–W–V complex was significantly higher than that of the MTC core, yet a quaternary M–M–W–V complex containing a truncated VRIMA^381–1486^ with N- and C-terminal disordered regions removed showed comparable activity to the MTC core (Supplementary information, Fig. S2). In parallel, we obtained the WTAP protein alone (residues 130–241, WTAP^130–241^) using the *Escherichia coli* expression system and determined the crystal structure of the WTAP (2.40 Å, Supplementary information, Table S1). Two molecules of WTAP (with the assigned density of residues 150–241, WTAP^150–241^) formed a symmetric parallel α-helical coiled-coil homodimer through hydrophobic interactions between two identical surface patches. Specifically, the side chains of residues M158, I165, L183, L204, I208, L211, V215, M218, I222, L225, L229 and L236 from the two WTAPs formed a hydrophobic interaction network (Fig. 1c; Supplementary information, Fig. S3a).

We further purified the METTL3–METTL14–WTAP^1–273^–VIRMA^381–1486^ complex for gradient fixation and cryo-EM analysis (Fig. 1b). Unexpectedly, in the cryo-EM structure, only WTAP^171–237^ and VIRMA^381–1292^ (with the stoichiometric ratio 2:1) and an unassigned density close to WTAP were observed (Supplementary information, Figs. S4, S5). The density for the rest of the quaternary complex was missing, suggesting high flexibility (Supplementary information, Table S2). The observed WTAP^171–237^–VIRMA^381–1292^ in the quaternary complex adopted a flag-and-pole-like architecture with dimensions of ~116 Å × 103 Å × 80 Å (Fig. 1d). Two WTAP^171–237^ molecules (designated WTAP_A_ and WTAP_B_) formed an asymmetric homodimer (the “flag pole”) while VIRMA^381–1292^ that contains 17 armadillo-like (ARML) repeats (designated ARML 1–17) formed a twisted α-solenoid-like superhelix (the “flag”). Of the ARML repeats, each of ARML 2–4, 6, 7, 9, 12, 15, and 17 consists of three helices while the rest are two-helix units. Two long helices in between of ARML 4–5 and of ARML 7–8 mediate the turning of the VIRMA α-solenoid, possibly accounting for the formation of the twisted architecture (Supplementary information, Figs. S6, S7).

The structure of the asymmetric WTAP^171–237^ dimer in the quaternary complex is similar to the crystal structure of the symmetric WTAP^150–241^ dimer with a root-mean-square deviation (RMSD) of 4.2 Å over 134 Cα atoms (Fig. 1e). However, compared to the WTAPs alone, VIRMA-bound WTAPs bent around the observed N-termini (around WTAP_A_ residue 188 and WTAP_B_ residue 185) and displayed a more compact and dense conformation with distinct intramolecular interactions (Fig. 1e). Hydrophobic interactions were found between WTAP_A_ and WTAP_B_ residues 180–236 through the side-chain interactions of WTAP_A_–WTAP_B_ residue pairs L183_A_–I180_B_ (L183 from WTAP_A_ and I180 from WTAP_B_), L187_A_–L183_B_, I208_A_–L204_B_, L211_A_–I208_B_, V215_A_–L211_B_, M218_A_–V215_B_, I222_A_–M218_B_, L225_A_–I222_B_, L229_A_–L225_B_, and L236_A_–L229_B_. WTAP K191_A_ and Q201_A_ side chains formed hydrogen bonds with Q190_B_ and Q201_B_ side chains, respectively. Hydrogen bond interactions were also observed between WTAP Q201_A_ side chain and K198_B_ main chain and between N205_A_ main chain and Q201_B_ side chain. These together contributed to the formation of the compact VIRMA-bound WTAP dimer that harbors a contiguous negatively charged surface patch involved in the interaction with VIRMA (Fig. 1e, f). Some of these residues involved in WTAP_A_–WTAP_B_ interactions (around residues 180–236) are also conserved in *Danio rerio* and *Drosophila melanogaster* WTAPs (Supplementary information, Fig. S3c).

Three intermolecular interaction interfaces, designated Interfaces 1, 2, and 3, could be observed between the WTAP^171–237^ dimer and VIRMA^381–1292^ with buried areas of ~773.9 Å^2^, ~1155.6 Å^2^, and ~101 Å^2^, respectively (Fig. 1d). Interface 1 is located in between WTAP_A_ (residues 205–224) and VIRMA (residues 452–660). Specifically, the side chains of WTAP N205_A_ and E216_A_ and VIRMA T660 and R545 formed hydrogen bonds, and E216_A_ and VIRMA R545 formed a salt bridge. V244_A_ main chain and VIRMA N452 side chain formed a hydrogen bond. Interfaces 2 and 3 are in between of WTAP_B_ (residues 177–200, 214–221) and VIRMA (residues 657–1030, 494–497). The side chains of WTAP Q177_B_, Q182_B_, E186_B_, K192_B_, E196_B_, E214_B_, and T221_B_ formed hydrogen bonds with the side chains of VIRMA D1030, D970, S965, S1025, N957, S497, and H494, respectively. WTAP S200_B_ and E203_B_ side chains formed hydrogen bonds with VIRMA A657 and M656 main chains, respectively (Fig. 1g; Supplementary information, Fig. S8a, b).

We further investigated the importance of the residues involved in the formation of Interfaces 1–3 via alanine substitutions and subsequent co-expression and purification assays (Supplementary information, Fig. S8). Specifically, Flag-tagged WTAP^1–273^ and His-tagged VIRMA^381–1486^ were co-expressed in the human embryonic kidney (HEK) 293F cells. The protein mixtures from the whole-cell extracts were subjected to western blot analysis against the tagged proteins and purification via anti-Flag beads. The single WTAP mutations (Q177A, Q182A, Q192A, E196A, E203A, N205A, or E216A) had little influence on WTAP–VIRMA^381–1486^ interaction, whereas the multi-alanine substitution mutant WTAP^Q177A/Q182A/K192A/E196A/E203A^ lost VIRMA^381–1486^-binding capacity (Supplementary information, Fig. S8c). The VIRMA^381–1486^ mutants containing individual N452A, T660A, N957A, or S1025A mutations retained binding to WTAP^1–273^, while the single alanine substitution of VIRMA^381–1292^ residue R545 or D970 abolished its interaction with WTAP^1–273^ (Supplementary information, Fig. S8d).

Despite that gel filtration analysis clearly indicates the presence of METTL3 and METTL14 in the METTL3–METTL14–WTAP^1–273^–VIRMA^381–1486^ quaternary complex, the cryo-EM density for the MTC core is largely missing in the cryo-EM structure, indicating that the MTC core is highly dynamic. This is also supported by previous reports that both METTL3 and METTL14 contain highly flexible intrinsically disordered regions (IDRs).^10^ Thus, we further conducted in vitro CXMS and AI-based prediction to model the structure of the quaternary M–M–W–V complex based on the solved structures. In total, we identified 227 (61 unique inter-subunit and 166 intra-subunit) bis(sulfosuccinimidyl) suberate (BS3)-crosslinked residue pairs with the false discovery rate (FDR) < 5% (Supplementary information, Fig. S9a). Notably, crosslinked pairs between WTAP coiled-coil and METTL3 N-terminal region (METTL3 residues 1–251, including METTL3–WTAP_A_ crosslinked pairs K13–K192 K132–K192, K241–K155, and K241–K160), VIRMA and the METTL14 N-terminal helix (NH, including METTL14–VIRMA crosslinked pair K38–K399), and VIRMA and MTC core catalytic center (including METTL3–VIRMA crosslinked pairs K576–K899, K576–K978, and K578–K899; METTL14–VIRMA crosslinked pair K148–K887) were identified (Supplementary information, Fig. S9b). No crosslinked pairs were identified between the METTL3 ZFD and METTL14 RGG domains with WTAP or VIRMA. The final structural model of the quaternary complex can account for all intermolecular crosslinks between METTL3/METTL14 core domains and the two regulatory subunits, except for two crosslinks involving flexible loop residues. In the highest-ranking model, the METTL3 N-terminal leader helix (LH) region (residues 1–34) was docked in close proximity to WTAP, accounting for the unassigned cryo-EM density (Fig. 1h, i; Supplementary information, Fig. S5c). This is in line with a previous report that the METTL3 LH is crucial for WTAP–METTL3 interaction.^11^ The rest of the METTL3 N-terminal region was also predicted to be close to WTAP. The N-terminus of METTL14 was docked close to VIRMA and potentially interacts with WTAP. Although the MTC core is likely dynamically positioned, its catalytic center was docked next to VIRMA with the catalytic center facing VIRMA ARML 9–10 (Fig. 1h, i). This model thus represents a possible resting state of the quaternary complex.

WTAP and VIRMA are important subunits of the m^6^A writer complex.^1,3^ We determined the structure of WTAP–VIRMA in the M–M–W–V complex at atomic resolution and modeled the quaternary complex structure using an AI-empowered integrative approach (Fig. 1j). Consistent with the previously reported adaptor role of WTAP,^3^ the observed WTAP dimer likely serves as a linker connecting VIRMA (and/or other MTC regulatory subunits) with the MTC core. VIRMA mediates preferential m^6^A mRNA methylation in 3′ UTR, but the exact mechanism is unknown.^6^ Like other ARML-containing proteins,^12^ the observed VIRMA superhelix contains extensive solvent-accessible surfaces that can accommodate RNA substrate^6^ and other regulator proteins.^6,13^ Moreover, in the model the VIRMA ARML 9–10 region (containing several positively charged residues) is close to the catalytic center of METTL3, suggesting a potential VIRMA–RNA interaction. During our manuscript revision, Su and colleagues also reported the structure of the regulatory subunit m^6^A-METTL-associated complex (MACOM) containing WTAP–VIRMA–ZC3H13–HAKAI.^14^ Our WTAP–VIRMA structure in the M–M–W–V complex is almost identical to the counterpart in the MACOM. Moreover, our AI-empowered integrative structural model indicates the interaction between the N-terminal helix of METTL3 and WTAP coiled-coil, and proximity between the MTC catalytic core and a VIRMA positively charged surface. As such, VIRMA N- and C-terminal disordered regions likely interact with the MTC core or substrate RNA, giving rise to the higher methyltransferase activity for the full-length VIRMA-containing quaternary complex. The two structural models provide a framework for future study on the molecular architecture and catalytic process of the m^6^A writer holocomplex, and offer new insights into potential therapeutic manipulation of m^6^A modification through the design of small molecules or peptides targeting WTAP–VIRMA interaction and modulating m^6^A catalytic specificity/activity.^15^ We anticipate that future structural and biochemical characterization of the complete m^6^A writer complex and its substrate-bound form will unveil the specificity and regulatory details underlying mRNA m^6^A methylation.

## Supplementary information

Supplemantary information

## Data Availability

Atomic coordinates of the WTAP^130–241^ has been deposited in the Protein Data Bank (PDB) under accession number 7YFJ. The cryo-EM density map for the human METTL3–METTL14–WTAP^1–273^–VIRMA^381–1486^ complex has been deposited in EM Database under the accession code EMD-33807. The corresponding atomic coordinates have been deposited in the PDB under accession code 7YG4. The CXMS data have been deposited to the ProteomeXchange Consortium PRIDE: PXD036144.
