# Supplementary material for: AI-empowered integrative structural characterization of m6A methyltransferase complex
Source: Cell Res. 2022 Nov 10;32(12):1124–7. doi: 10.1038/s41422-022-00741-8 (PMC9715571; doi:10.1038/s41422-022-00741-8)
Supplement: Supplementary file 1 — Supplemantary information [file 41422_2022_741_MOESM1_ESM.pdf]

AI-empowered integrative structural characterization of m6A methyltransferase complex

Xuhui Yan[1,3], Kai Pei[2,3], Zeyuan Guan[1,3], Feiqing Liu[1], Qiang Wang[1], Junjun Yan[1], Xiaohuan Jin[1], Mengjun Hou[1], Chun Tang[2,*], and Ping Yin[1,*]

[1]National Key Laboratory of Crop Genetic Improvement, Hubei Hongshan Laboratory, Huazhong Agricultural University, Wuhan 430070, China.

[2]Beijing National Laboratory for Molecular Sciences, College of Chemistry and Molecular Engineering, PKU-Tsinghua Center for Life Sciences, Center for Quantitative Biology, Peking University, Beijing 100871, China.

*Correspondence:
Chun Tang (Tang_Chun@pku.edu.cn) or Ping Yin (yinping@mail.hzau.edu.cn)

[3]These authors contributed equally.

Supplementary information

Material and Methods

Molecular cloning and protein expression

Full-length METTL3, METTL14, WTAP, and VIRMA were amplified from the *Homo sapiens* cDNA library. The truncated genes were subcloned using the standard polymerase chain reaction (PCR) method. All site-directed mutagenesis of WTAP and VIRMA was carried out using the Fusion PCR method.

For the cryo-EM sample preparation and the methyl transfer assay, the DNAs of human METTL3 and METTL14, WTAP (the $WTAP^{1-273}$ for cryo-EM sample preparation; the full-length WTAP for the methyl transfer assay), and $VIRMA^{381-1486}$ were subcloned into pFastBac dual. Both METTL14 and WTAP were expressed with an N-terminal His-tag. For the co-expression coupled purification assay, the genes WTAP and VIRMA were subcloned into a modified pFastBac1 vector, fused with a His-tag at the N-terminus. METTL3 and METTL14 were subcloned into pFastBac dual with a Strep-tag and His-tag at N-terminus, respectively. The baculoviruses were generated in Sf9 cells with the bac-to-bac system (Invitrogen). The proteins were co-expressed in Sf9 cells at 27 °C for 60 h before harvesting.

For the crystallization trials, the $WTAP^{130-241}$ was subcloned into pET21b (Novagen) and fused with a His-tag at the C-terminus. The plasmid was transformed into BL21 (DE3). One-liter lysogeny broth medium supplemented with 100 mg/mL ampicillin was inoculated with a transformed bacterial pre-

culture and shaken at 37 °C until the optical density at 600 nm reached 1.0. After being induced with 0.2 mM isopropyl-β-D-thiogalactoside (IPTG) and growing at 16 °C for 14 h before harvesting.

For the structure-guided mutagenesis analysis, Flag-tag $WTAP^{1-273}$ (plasmid 1) and His-tag $VIRMA^{381-1486}$ (plasmid 2) were also subcloned into the pMlink vector with a C-terminal tag, respectively. The Expi293F™ (Invitrogen) cells were cultured in SMM 293TI medium (Sino Biological Inc.) at 37 °C under 5% CO_2 in a ZCZY-CS8 shaker (Shanghai Zhichu Instrument co., Ltd.) and diluted into 2.0×10^6 cells/mL for further transfection. For 30 mL cell culture, 30 μg plasmid 1 and 30 μg plasmid 2 were pre-incubated with 180 μg linear polyethylenimines (PEIs) (Polysciences) in 2 mL fresh medium for 20 min. The transfection was initiated by adding the mixture to the diluted cell culture. Transfected cells were cultured for 48 h before harvesting.

Protein purification

For the cryo-EM sample preparation and mRNA methyltransferase assay, the Sf9 cells were harvested by centrifugation at 2000× *g* for 15 min and homogenized in ice-cold lysis buffer (25 mM Tris-HCl, pH 8.0 and 150 mM NaCl) with 1 mM phenylmethanesulfonyl-fluoride (PMSF). The supernatants were loaded onto Ni-NTA resin (Qiagen) and washed three times with lysis buffer containing 10 mM imidazole. Elution was performed in Elution buffer (25 mM Tris-HCl, 250 mM Imidazole, pH 8.0 and 2 mM Dithiothreitol (DTT)), and applied to a Source Q10/100 column (GE Healthcare), followed by a gradient NaCl elution (up to 1 M) in 25 mM Tris-HCl (pH 8.0). Target proteins were further purified on a Superose 6 increase 10/300 GL column (GE Healthcare) equilibrated with SEC buffer (50 mM

HEPES, pH 7.5, 50 mM NaCl, and 50 μM $MgCl_2$).

To acquire the METTL3-METTL14-WTAP-VIRMA complex, METTL3-METTL14 protein and WTAP-VIRMA protein were mixed at a molar ratio of 1:0.9 at 4 °C for 30 min. Then the mixture was applied to Superose 6 increase 10/300 column equilibrated with SEC buffer. The METTL3-METTL14-WTAP-VIRMA$^{381-1486}$ and the METTL3-METTL14-WTAP-VIRMA were used for the methyl transfer assay. To prepare the sample for the cryo-EM, the peak fractions of METTL3-METTL14-WTAP$^{1-273}$-VIRMA$^{381-1486}$ were further treated by Gradient fixation (GraFix[1]). In detail, low buffer (SEC buffer containing 10% v/v glycerol) was layered on top of an equal volume of freshly prepared high buffer (SEC buffer containing 25% v/v glycerol and 0.05% glutaraldehyde) in a 12.5 mL tube before cooling on ice. Centrifugation was performed at 33,000 rpm in a Beckman SW40Ti swinging bucket rotor for 18 h at 4 °C. GraFix peak fractions were collected by gradient fractionator (Biocomp), quenched with 100 mM Tris-HCl (pH 8.0), desalted (Hitrap desalting, GE Healthcare) into 25 mM imidazole, pH 8.0, 150 mM NaCl, 5 mM DTT and concentrated to 0.4 mg/mL.

For the crystallization trials, the bacterial pellet was collected and homogenized in a lysis buffer containing 1 mM PMSF. After centrifugation at 14,000 rpm at 4 °C, the supernatant was loaded onto Ni-NTA resin; washed three times with lysis buffer containing 15 mM imidazole, and eluted with Elution buffer. The eluted protein was applied to a Source Q10/100 column. Target proteins were subjected to a Superose 6 10/300 GL column (GE Healthcare), which was equilibrated with a buffer containing 25 mM Tris-HCl, pH 8.0, 150 mM NaCl, and 5 mM DTT.

For the co-expression coupled purification assay and structure-guided mutagenesis analysis, the cells were resuspended in 1 mL lysis buffer containing 1 mM PMSF, and lysed by repeated freeze-thaw using liquid nitrogen. After ultracentrifugation, the supernatant was loaded onto a Strep-affinity or Flag-affinity column, washed using lysis buffer, and eluted using a lysis buffer containing 3 mM desthiobiotin, or 0.25 mg/mL Flag peptide (GenScript), respectively. The expression of WTAP and VIRMA were verified by western blot.

Crystallization

The WTAP$^{130-241}$ protein was crystallized by using the hanging-drop vapor diffusion method at 18 °C, and 1 μL of the sample was mixed with an equal volume of reservoir solution. Diamond-shaped crystals appeared overnight from a reservoir solution containing 0.1 M MES (2-(N-morpholino) ethanesulfonic acid), pH 6.0, 160 mM magnesium nitrate, 21 mM sodium bromide, 8% PEG6000 and 11% glycerol. After 48 h of growth, the crystal ceased growing and was flash-frozen in liquid nitrogen. The cryoprotection buffer including 20% v/v ethylene glycol and 10% w/v NDSB-201 added to the reservoir solution. The WTAP$^{130-241}$ crystal diffracted beyond 2.40 Å at the Shanghai Synchrotron Radiation Facility (SSRF) beamline BL17U1 (ref. 2).

Crystal structure determination

The dataset was collected at the SSRF beamline BL17U1 and processed with the HKL3000 (ref. 3). Further processing was performed with the CCP4 suite[4]. Data collection and structural refinement statistics are summarized in Table S1. The structure of the WTAP$^{130-241}$ was solved by molecular

replacement (MR) using the prediction structure from AlaphaFold2 (ref. 5) as the search models using the program PHASER[6]. The structure was manually and iteratively refined with PHENIX[7] and COOT[8]. All figures representing structures were prepared with PyMOL.

Sample preparation and cryo-EM data collection

For cryo-EM data acquisition of the METTL3-METTL14-WTAP$^{1\text{-}273}$-VIRMA$^{381\text{-}186}$ complex, right before grid preparation the β-octyl glucoside was added to a final concentration of 0.05%, 3.5 μL sample were deposited onto a freshly glow-discharged (Thermo Fisher, 20 mA, 120 s) holey carbon grid (Quantifoil R1.2/1.3, Au 300 mesh) and plunged into liquid ethane using an FEI Virobot Mark IV after blotting for 3.5 s with blot force 0, Whatman 597 filter paper at 4 °C and 100 % humidity. Each grid was screened using a Thermo Scientific™ Glacios™ Cryo-EM at 200 keV. Cryo-EM data were collected on a Titan Krios TEM operated at 300 keV and equipped with a K3 Summit direct detector (Gatan) positioned to post a GIF quantum energy filter (slit width 20 eV). Automated data acquisition was carried out using EPU in super-resolution mode at a magnified pixel size of 0.85 Å, with defocus values ranging from −1.0 to −1.5 mm. The total exposure time was set to 2.51 s with 40 frames, resulting in an accumulated dose of about 55.1 e^- per $Å^2$.

Cryo-EM data processing, model building and refinement

The schematic of the data processing pipeline is shown in Fig. S4c. About 915,354 particles from 2040 micrographs were automatically picked using the cryoSPARC blob picker[9]. After two-dimensional classification, a total of 890,453 good particles were selected and subjected to several cycles of three-

dimensional classification in cryoSPARC[9]. 197,685 particles belonging to the best class were selected; this is followed by nonuniform refinement and local refinement. The METTL3-METTL14-WTAP-VIRMA complex yielded a cryo-EM density with an estimated resolution of 3.10 Å based on gold standard Fourier shell correlation[10].

The atomic model for the METTL3-METTL14-WTAP-VIRMA complex was built in COOT[11] and refined with PHENIX[12]. The structure of the METTL3-METTL14-WTAP-VIRMA was validated through the examination of Molprobity[13] scores and the Ramachandran plots (Table S2).

RNA sample preparation and the methyl transfer assay

The specific RNA (5′-UACACUCGAUCUGGACUAAAGCUGCUC-3′)[14], and the nonspecific RNA (5′- UACACUCGAUCUUUUUUAAAGCUGCUC-3′, as negative control) were used in the methyl transfer assay. RNA samples were synthesized using the ABI-3400 Synthesizer with phosphoramidites as previously described[15].

Reactions were carried out in duplicates with a 10 μL reaction mixture containing [ssRNA]=1 oligos, [SAM]=1 μM, [E]=1 μM in SEC buffer at 25 °C for 20 min. Reactions were terminated by the addition of trifluoroacetic acid (TFA) to a final concentration of 0.1% (v/v) and an 8-μL mixture was transferred to a Half-Area 384-well plate. The activity was measured using an MTase-Glo™ Methyltransferase Assay kit in which the reaction by-product SAH is converted into ATP in a two-step reaction and ATP can be detected through a luciferase reaction[16]. The luminescence signal was measured by a TECAN

infinite M200 (TECAN).

In vitro cross-linking

The cross-linking agent bis(sulfosuccinimidyl) suberate (BS3, from Thermo Fisher) was prepared at a concentration of 100 mM in DMSO. BS3 was added to the quaternary protein complex (4.8 μM) to a final concentration of 1.67 mM. The reaction was performed at 30 ºC for 30 min and then quenched by the addition of 20 mM Tris-HCl (pH 7.5). The experiments were triplicated with three parallel samples.

Mass spectrometry and data analysis.

The cross-linked protein sample was precipitated with 6 volume pre-chilled acetone at 4 ℃ and centrifuged for 20 min to remove supernatant. The precipitate sample was dissolved in 8 M urea and 0.1 M Tris-HCl (pH 8.5), reduced with 5 mM DTT at 25 ℃ for 10 min, and alkylated with 10 mM iodoacetamide in dark for 15 min. Subsequently, 3 volumes of Tris-HCl (pH 8.5) were added to dilute the sample, which also contained 1 mM $CaCl_2$ (to repress Chymotrypsin activity) and 20 mM methylamine (to reduce the modification of the Carbamate modification at the N-terminus of the peptide segment). Trypsin digestion was carried out at 37 °C overnight with sequencing grade modified trypsin (Promega, mass ratio = 1:20). The reaction was quenched with trifluoroacetic acid to a final concentration of 5%.

Trypsin-digested peptides were purified with C18 Spin Tips (Thermo Fisher Scientific) and were analyzed in the Orbitrap Fusion Lumos mass spectrometer (Thermo Fisher Scientific) coupled to an

EASY-nLC 1200 liquid chromatography system, with a 75 µm, 2 cm Acclaim PepMapTM 100 column. The peptides were eluted over a 65 min linear gradient from 95% buffer A (water with 0.1% Formic acid) to 35% buffer B (acetonitrile with 0.1% Formic acid) with a flow rate of 200 nL/min. Each full MS scan (Resolution = 70,000) was followed by 15 data-dependent MS2 (Resolution = 17,000), with HCD (higher-energy-collisional-dissociation) and an isolation window of 1.6 m/z. The normalized collision energy was set to 30. Precursors of charge state ≤ 3 were collected for MS2 scans in the enumerative mode; precursors of charge state 3–6 were collected for MS2 scans in cross-link discovery mode. Monoisotopic precursor selection was enabled, and a dynamic exclusion window was set to 30 s.

The cross-linking data were analyzed by pLink2 (ref. 17). The following search parameters were used: MS1 accuracy of ±20 ppm; MS2 accuracy = ±20 ppm; enzyme = trypsin (with full tryptic specificity but allowing up to three missed cleavages); crosslinker = BS3 (with an assumed reaction specificity for lysine and protein N termini); fixed modifications = carbamidomethylation on cysteine; variable modifications = oxidation on methionine, hydrolyzed/aminolyzed BS3 from reaction with ammonia or water on a free cross-linker end. The identified candidates have filtered these parameters: false discovery rate (FDR) < 5%, supervised vector machine or SVM score < 10^{-2}, and abundance or peptide-spectrum matches (PSMs) ≥ 3. The experimental cross-links were illustrated with Crosslink-viewer[18]. Only cross-links that were observed in at least two biological repeat experiments were used for structural modeling of the quaternary complex.

AI-based structure prediction

Each experimental cross-link was converted to distance restraints. They were applied to the Cα atoms of the cross-linked residues with an upper distance bound of 26 Å (a 2-Å padding was added to account for local flexibility[19]). However, due to the limited reactivity of BS3, multiple binding modes between proteins have to be invoked to account for the intermolecular cross-linking data[20, 21]. On the other hand, due to the sparsity of the restraints, each binding mode cannot be effectively validated.

We used AlaphaFold2 (version 2.2) multimer[5] to build the structural models of the complex. To narrow the search space, only the structural models of binary complexes were predicted. Prediction of the binary complex was further sped up by including only the N terminal domains of METTL3 (with WTAP) and METTL14 (with VIRMA), to which the majority of the intermolecular cross-links were identified. On the other hand, VIRMA residues D342-D1292 and WTAP residues T148-D237 were included in AlaphaFold2 multimer prediction. The initial model of the quaternary complex was further constructed with the incorporation of the known binary complex structures of METTL3-METTL14 catalytic domain and METTL3 ZFD[15, 22] and VIRMA-WTAP (this study).

The position of the METTL3-METTL14 catalytic domain was further optimized by incorporating the cross-link restraints to residue K398 near the C-terminus of METTL14. Since AlphaFold2 gave a low-confidence prediction to the flexible linker residues[23], database potential[24] and a weak radius of gyration potential[25] were further incorporated for conformation optimization and packing against the structured portions.

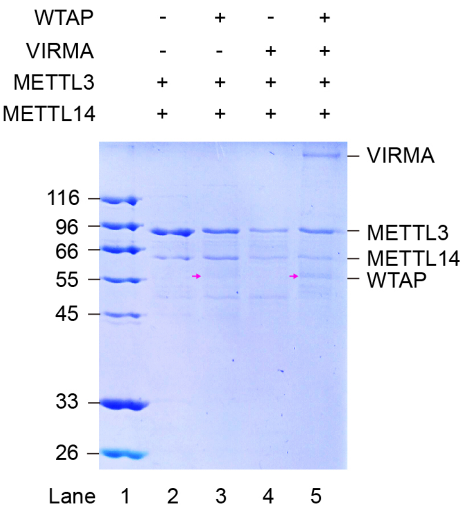

Fig. S1 The interaction relationship between the subunits of M-M-W-V complex. Co-express Strep-METTL3/His-METTL14, Strep-METTL3/His-METTL14/His-WTAP, strep-METTL3/His-METTL14/His-VIRMA and Strep-METTL3/His-METTL14/His-WTAP/His-VIRMA coupled Strep-tag purification. Red arrow indicates the WTAP.

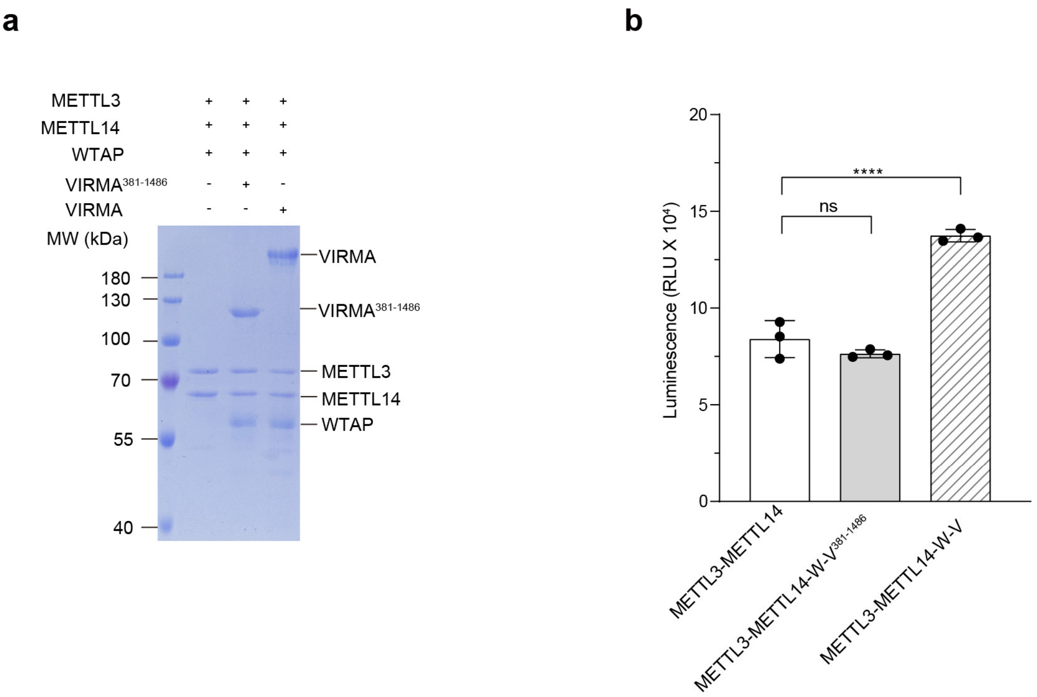

Fig. S2 Relative methyl transfer activity of the METTL3-METTL14, METTL3-METTL14-WTAP-VIRMA$^{381-1486}$ and METTL3-METTL14-WTAP-VIRMA complexes. a SDS-PAGE of the METTL3-METTL14, METTL3-METTL14-W-V$^{381-1486}$ and METTL3-METTL14-W-V complexes. **b** The methyl transfer activity of MTCs, corresponding to **a**. [ssRNA]= 1 μM oligos, [SAM]=1 μM, [E]=1 μM. The error bars represent the SD of three independent measurements.

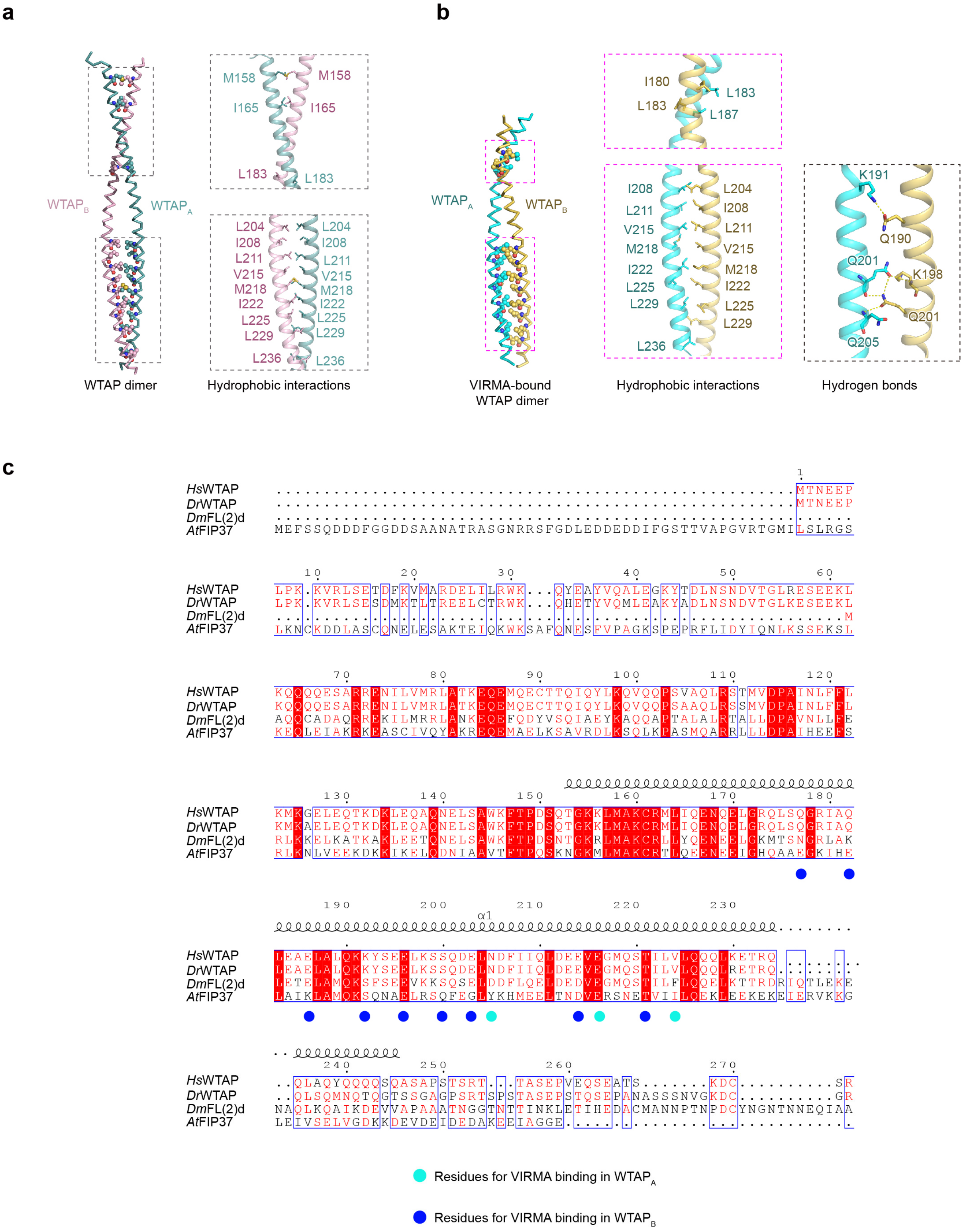

Fig. S3 The interactions between WTAP$_A$ and WTAP$_B$. a In the crystal structure, two molecules of WTAP forms a symmetric parallel alpha-helical coiled-coil with hydrophobic interactions. **b** VIRMA-bound WTAP forms an asymmetric coiled-coil with hydrophobic interactions and hydrogen bonds. **c** Sequence alignment of WTAP in *Homo sapiens*, *Danio rerio*, *Drosophila melanogaster* and

*Arabidopsis thaliana***.** The alignment was generated using the MultAlin and ENDscript programs. Secondary structural elements are shown above. The residues involving in WTAP-VIRMA interaction were illustrated by solid squares.

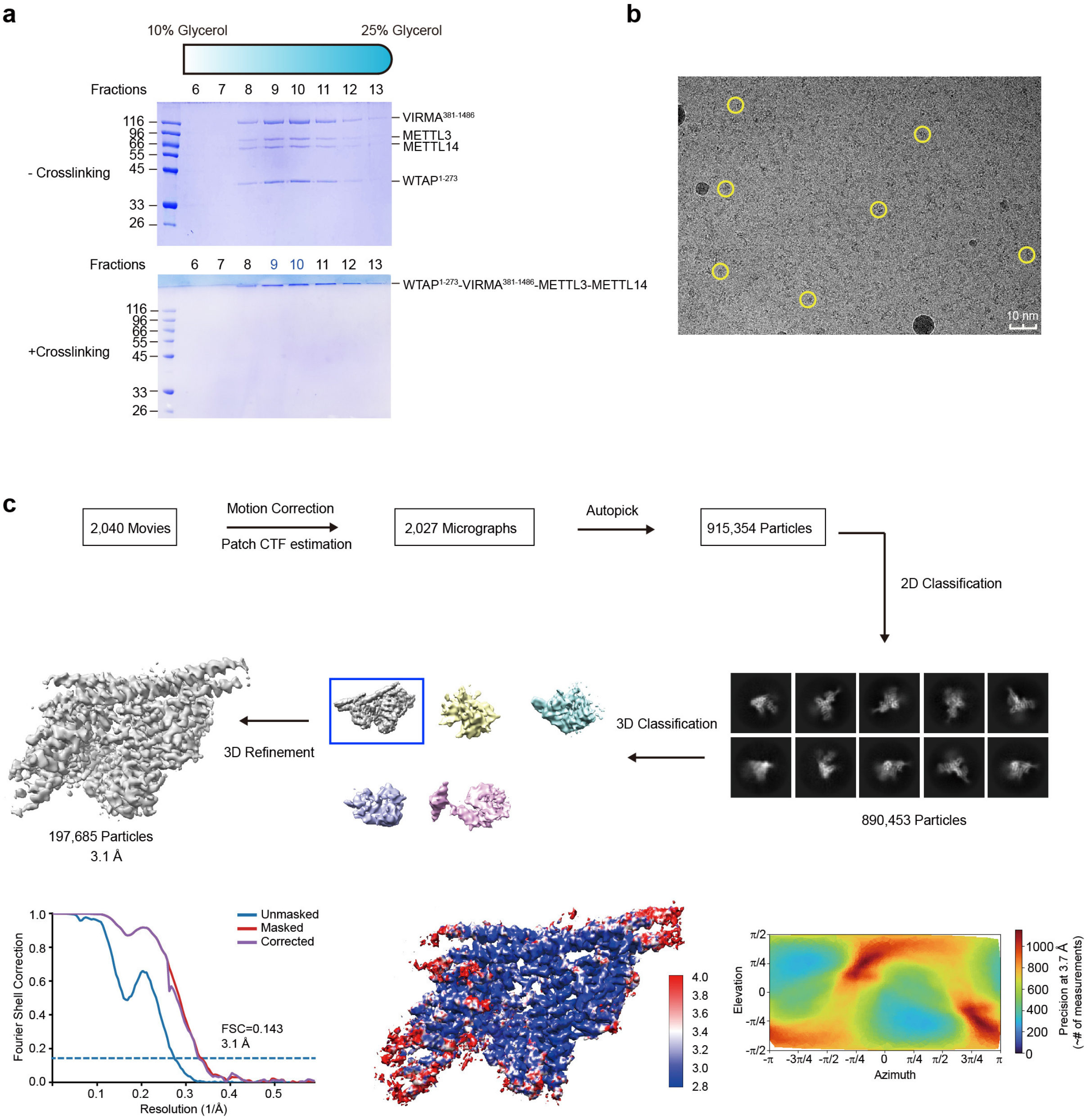

Fig. S4 Sample preparation for cryo-EM experiments and the data processing of the METTL3-METTL14-WTAP$^{1-273}$-VIRMA$^{381-1486}$ complex. **a** Representative SDS-PAGE showing fractions taken across glycerol gradient prepared without/with cross-linking agent glutaraldehyde. Fractions 9 and 10 were pooled for cryo-EM grid preparation. **b** Representative cryo-EM micrograph. **c** Flowchart for cryo-EM data processing of the human METTL3-METTL14-WTAP$^{1-273}$-VIRMA$^{381-1486}$ complex. The FSC curves of the final refined models of the METTL3-METTL14-WTAP$^{1-273}$-VIRMA$^{381-1486}$ complex. Local resolutions are color-coded for the METTL3-METTL14-WTAP$^{1-273}$-VIRMA$^{381-1486}$ complex. The highest resolution of the EM maps reaches 2.80 Å.

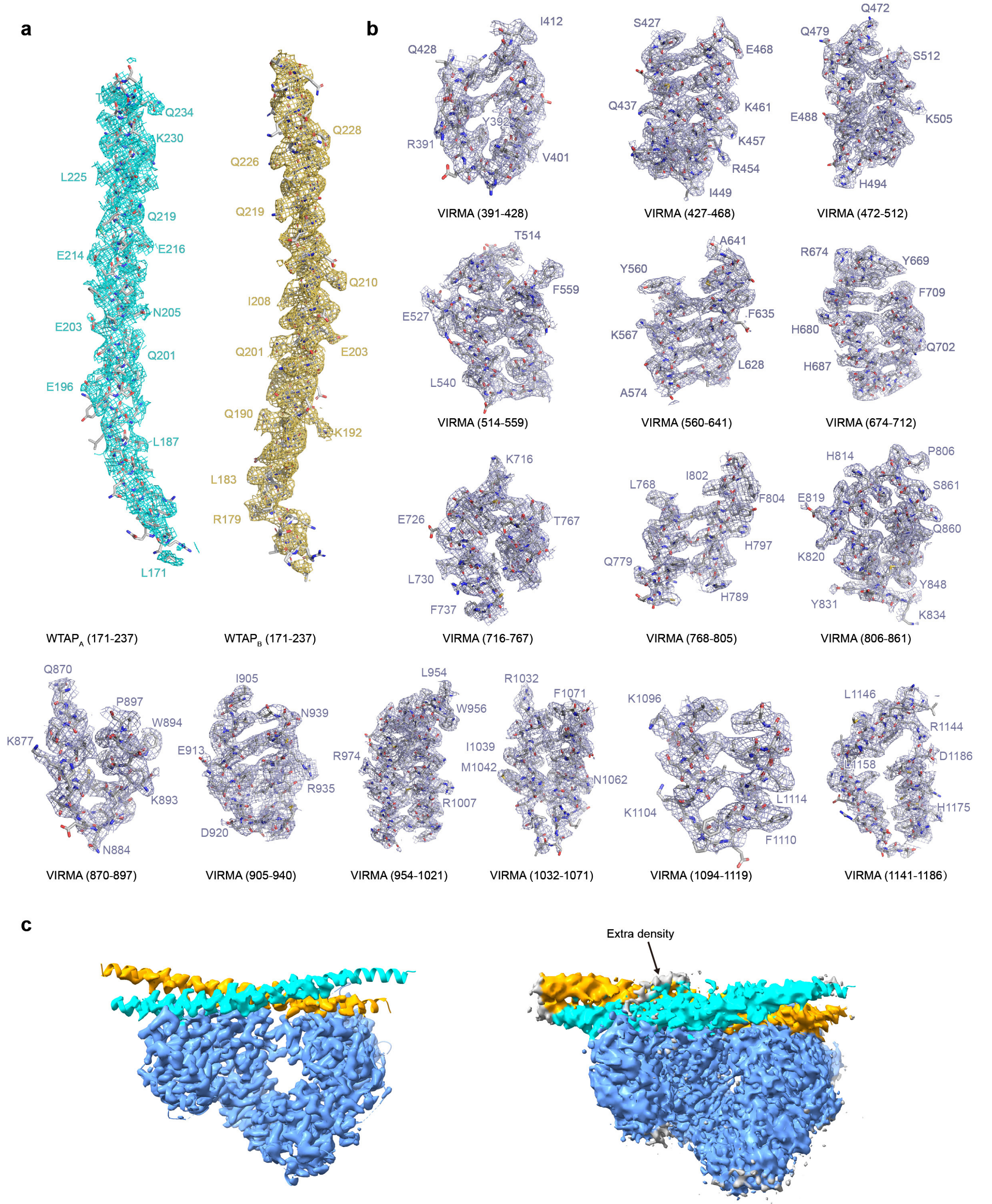

Fig. S5 The electron microscopy maps for the METTL3-METTL14-WTAP$^{1-273}$-VIRMA$^{381-1486}$ complex. a The electron microscopy maps for WTAP$_A$ and WTAP$_B$. **b** The electron microscopy maps for ARMLs. **c** High and low threshold of METTL3-METTL14-WTAP$^{1-273}$-VIRMA$^{381-1486}$ complex. Arrow indicates the extra density.

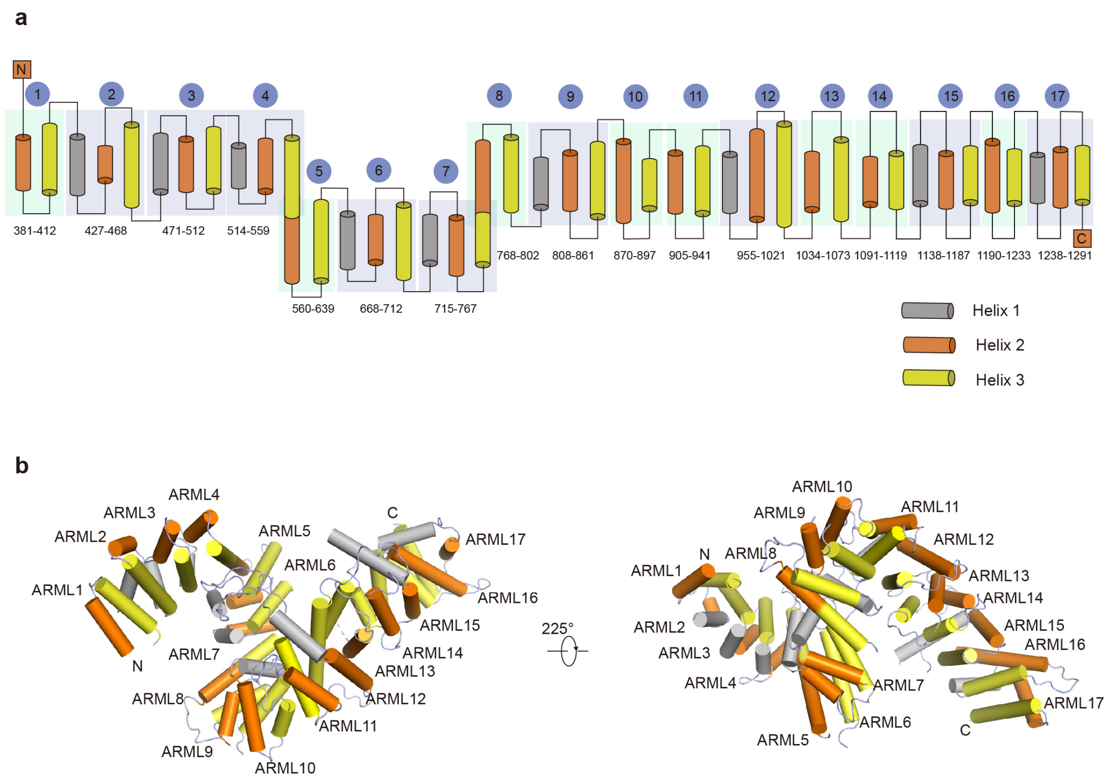

Fig. S6 Characterization of the structure of ARMLs. a The topological diagram of the ARMLs. VIRMA$^{381\text{-}1292}$ is mainly composed of alpha-helices, including 17 ARMLs. Each alpha-helix is ~12 amino-acid long, consisting of two long helices which are 26 and 29 amino-acid, respectively. ARML contains two or three helices, H1 (gray), H2 (orange), and H3 (yellow). ARML 2-4, 6, 7, 9, 12, 15 and 17 consists of three helices while the rest are two-helix units. Two long helices in between of ARML 4-5 and ARML 7-8 mediate the turning of the VIRMA alpha-solenoid. **b** Overall structure of the VIRMA$^{381\text{-}1292}$ is represented as cartoon cylindrical helices, with the ARML repeats labeled ARML1 to 17.

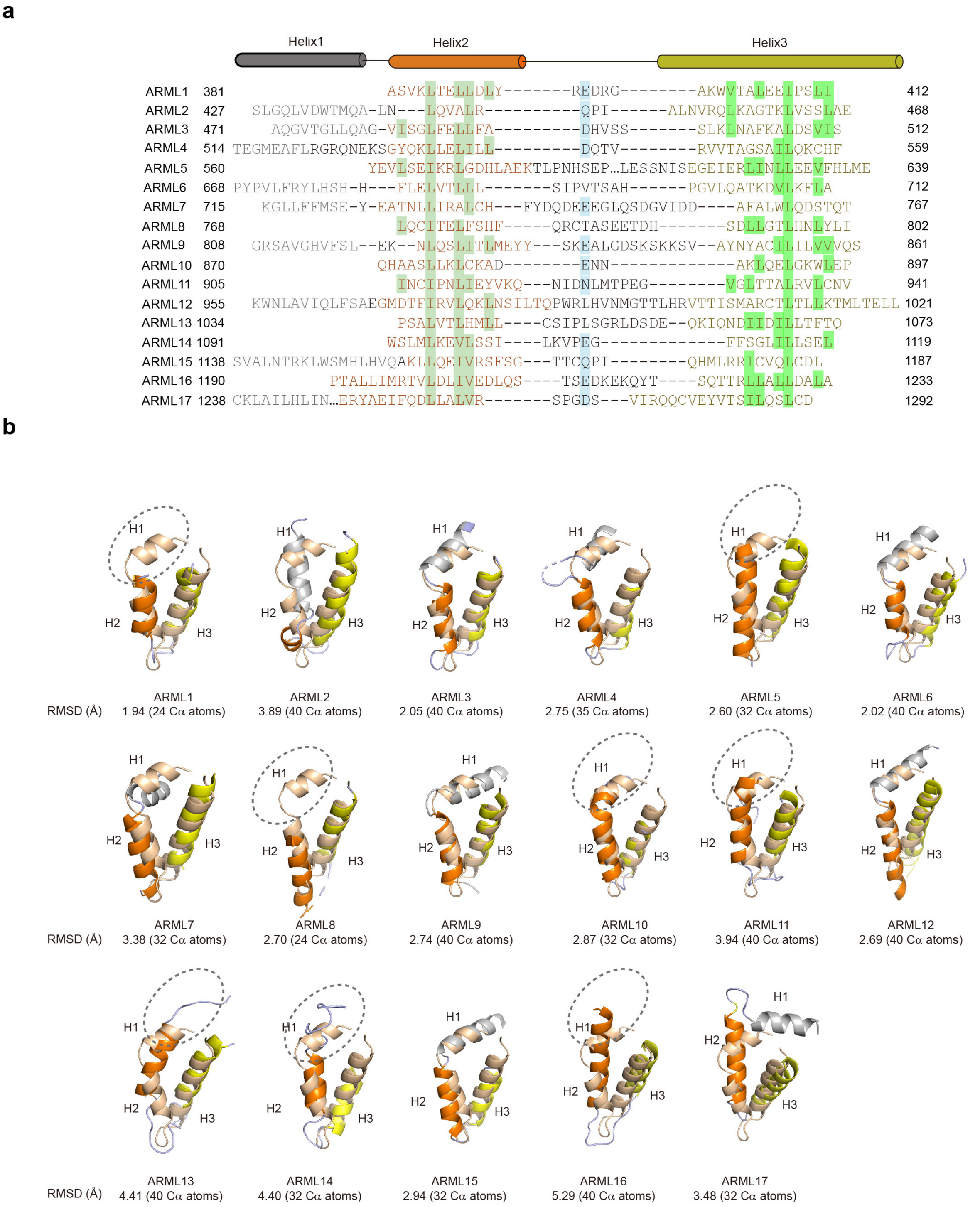

Fig. S7 Sequence and structure alignment of the seventeen ARMLs of VIRMA. a Sequence alignment of the seventeen ARMLs of VIRMA. Conserved residues that define the ARML consensus motif are highlighted in reseda and green. **b** Superposition of the VIRMA's ARMLs and the β-Catenin's ARM 2 (wheat, PDB 1BK6). Residues forming H1, H2, and H3 helices of each ARML are highlighted in gray, orange, and yellow, respectively.

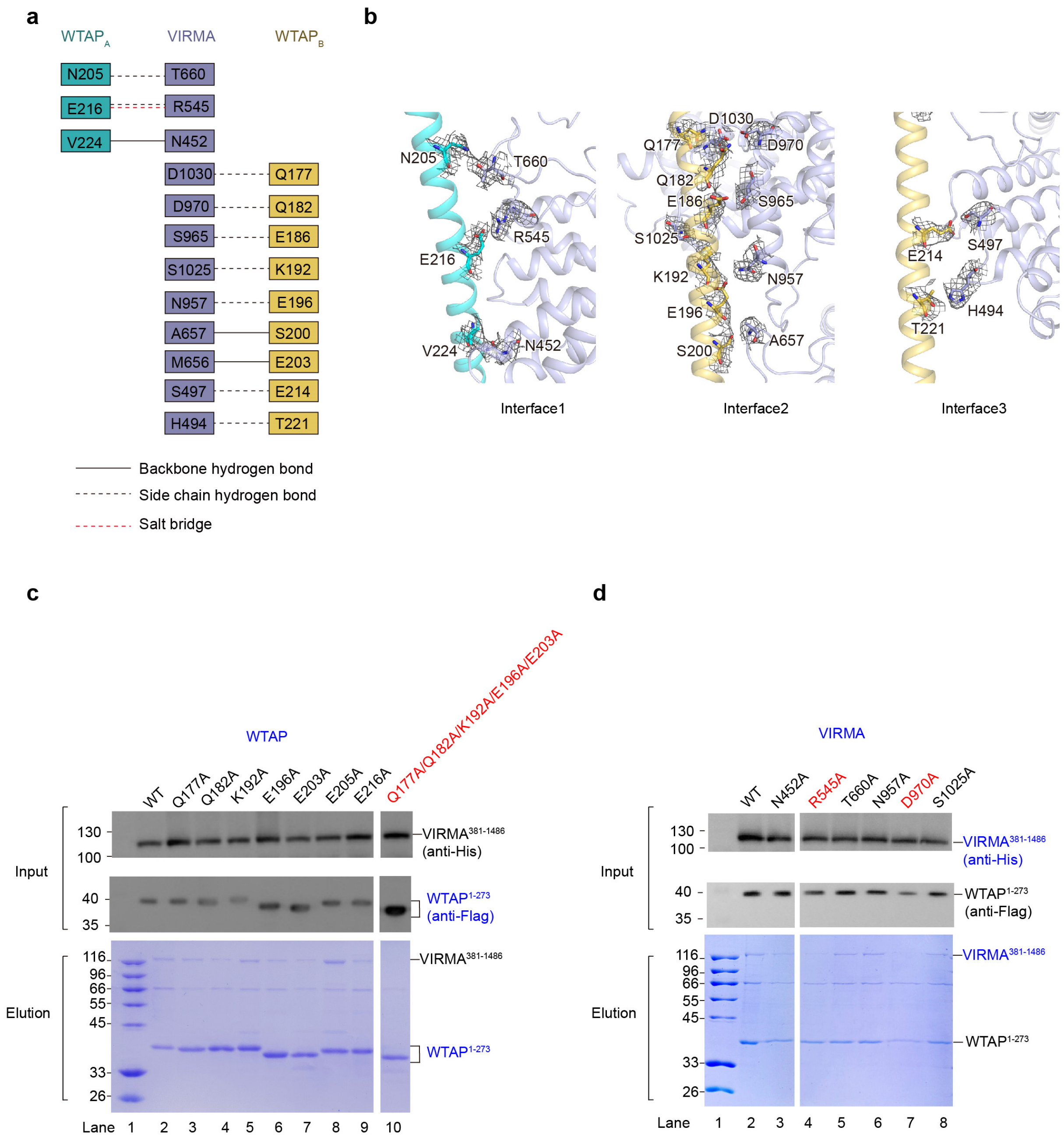

Fig. S8 Structure-guided mutagenesis analysis of WTAP-VIRMA interaction interfaces. a Schematic representation of interaction networks between WTAP and VIRMA. Residues in $WTAP_A$, $WTAP_B$, and VIRMA involved in the interactions are shown in cyan, yellow orange and light blue rectangles, respectively. **b** The density fitting of the inter-subunit interaction interfaces between WTAPs and VIRMA. **c**, **d** Co-express $WTAP^{1-273}$ muts-Flag/$VIRMA^{381-1486}$-His (**c**) and $WTAP^{1-273}$-Flag/$VIRMA^{381-1486}$ muts-His (**d**) coupled Flag-tag purification. Residues critical for complex formation are colored in red.

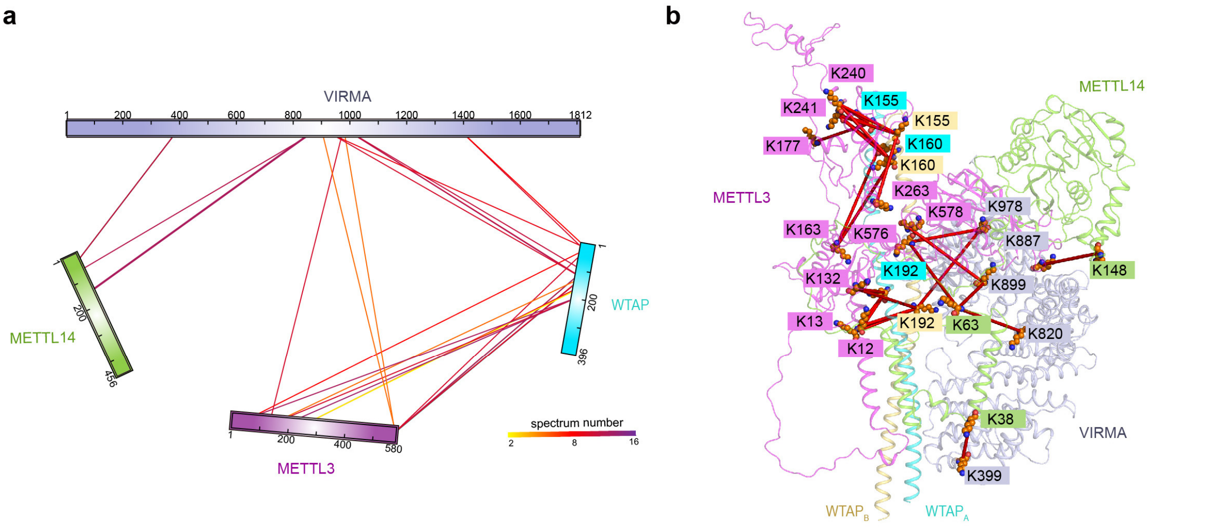

Fig. S9 CXMS analysis of METTL3-METTL14-WTAP-VIRMA$^{381\text{-}1486}$ complex architecture. a Inter-subunit crosslinks MTC, METTL3, METTL14, WTAP and VIRMA$^{381\text{-}1486}$. The numbers of the corresponding spectra of each cross-link were indicated by the color of the line. Cross-links were filtered by requiring FDR < 0.05 at the spectra level, SVM-value < 1×10^{-2} and spectral counts ≥ 3. **b** The inter-subunit BS3-crosslinked residue pairs. Residues in METTL3, METTL14, $WTAP_A$, $WTAP_B$ and VIRMA involved in the crosslinked pairs are shown in magenta, chartreuse, cyan, yellow and light blue rectangles, respectively.

Table S1 Statistics of crystal data collection and refinement of the human WTAP.

	$WTAP^{130\text{-}241}$ PDB: 7YFJ
Space group	$P6_122$
Cell dimensions	
a, b, c (**Å**)	62.75, 62.75, 336.35
α, β, g (°)	90, 90, 120
Number of molecules in ASU	2
Wavelength (**Å**)	0.97915
Resolution (**Å**)	45~2.40(2.49~2.40)
*R*merge (%)	9.9(97.8)
*R*pim (%)	3.1(30.0)
I/*σ* I σ	18.8(2.4)
Completeness (%)	99.5(99.6)
Number of measured reflections	331,188
Number of unique reflections	16,547
Redundancy	20.0(20.7)
Wilson B factor (**Å**^{2})	55.66
R_{work}/R_{free} (%)	21.95/23.98
Number of atoms	1656
Protein	1558
Main chain	756
Side chain	802
Water	84
Other entities	14
Average B value (**Å**^{2})	65.2
Protein	65.3
main chain	87.7
side chain	80.6
Water	86.4
Other entities	132.6
R.m.s. deviations	
Bond lengths (**Å**)	0.008
Bond angle (°)	0.925
Ramachandran plot statistics (%)	
Most favorable	98.3
Allowed	1.7
Disallowed	0

Table S2 Statistics of cryo-EM data collection and refinement of the METTL3-METTL14-WTAP-VIRMA complex.

	METTL3-METTL14-WTAP$^{1-273}$-VIRMA$^{381-1486}$<br>PDB: 7YG4<br>EMBD: EMD-33807
**Data collection and processing**	
Microscope	Krios
Voltage (kV)	300
Camera	Gatan K3
Magnification	105,000
Pixel size at detector (Å/pixel)	0.85
Total electron exposure (e^{-}/Å^{2})	55.1
Frames collected during exposure (no.)	40
Defocus range (μm)	-1.0~-1.5
Automation software	EPU
Micrographs collected (no.)	2,040
Micrographs used (no.)	2,027
Total extract particles (no.)	915,354
**For each reconstruction**	
Refined particles (no.)	197,685
Final particles (no.)	197,685
Point group	C1
Resolution (global, Å)	
FSC 0.5 (unmasked/masked)	5.9/3.6
FSC 0.143 (unmasked/masked)	3.7/3.0
Resolution range (local, Å)	
Map sharpening B factor (Å^{2})	96.7
Map sharpening methods	Half-maps correlation
**Model composition**	
Protein	953
Ligands/DNA/RNA	0
**Model refinement**	
Refinement package	PHENIX

- real or reciprocal space	Real Space
- resolution cutoff	3.1
Model-Map scores	
- CC	0.85
B factors (Å^2)	
Protein residues	75.34
Ligands/DNA/RNA	0
R.m.s. deviations from ideal values	
Bonds length (Å)	0.003
Bond angles (°)	0.584
**Validation**	
MolProbity score	1.60
CaBLAM outliers	1.64
Clashscore	7.09
Poor rotamers (%)	0
C-beta deviations	0
EMRinger score (if better than 4 Å resolution)	1.84
Ramachandran Plot	
Favored (%)	96.68
Outliers (%)	0

References

1 Kastner B, Fischer N, Golas MM et al. GraFix: sample preparation for single-particle electron cryomicroscopy. *Nat Methods*. **5**, 53-55 (2008).

2 Wang Z, Pan Q, Yang L et al. Automatic crystal centring procedure at the SSRF macromolecular crystallography beamline. *J Synchrotron Radiat*. **23**, 1323-1332 (2016).

3 Otwinowski Z, Minor W. Processing of X-ray diffraction data collected in oscillation mode. *Methods Enzymol*. **276**, 307-326 (1997).

4 Collaborative Computational Project, Number 4. The CCP4 suite: programs for protein crystallography. *Acta Crystallogr D Biol Crystallogr*. **50**, 760-763 (1994).

5 Evans R, O'Neill M, Pritzel A et al. Protein complex prediction with AlphaFold-Multimer. *bioRxiv*. DOI: https://doi.org/10.1101/2021.10.04.463034

6 McCoy AJ et al. Phaser crystallographic software. *J Appl Crystallogr*. **40**, 658-674 (2007).

7 Adams PD, Grosse-Kunstleve RW, Hung LW et al. PHENIX: building new software for automated crystallographic structure determination. *Acta Crystallogr D Biol Crystallogr*. **58**, 1948-1954 (2002).

8 Emsley P, Cowtan K. Coot: model-building tools for molecular graphics. *Acta Crystallogr D Biol Crystallogr*. **60**, 2126-2132 (2004).

9 Punjani A, Rubinstein JL, Fleet DJ, Brubaker MA. cryoSPARC: algorithms for rapid unsupervised cryo-EM structure determination. *Nat Methods*. **14**, 290-296 (2017).

10 Rosenthal PB, Henderson R. Optimal determination of particle orientation, absolute hand, and contrast loss in single-particle electron cryomicroscopy. *J Mol Biol*. **333**, 721-745 (2003).

11 Emsley P, Lohkamp B, Scott WG, Cowtan K. Features and development of Coot. *Acta Crystallogr D Biol Crystallogr*. **66**, 486-501 (2010).

12 Adams PD, Afonine PV, Bunkoczi G et al. PHENIX: a comprehensive Python-based system for macromolecular structure solution. *Acta Crystallogr D Biol Crystallogr*. **66**, 213-221 (2010).

13 Davis IW, Leaver-Fay A, Chen VB et al. MolProbity: all-atom contacts and structure validation for proteins and nucleic acids. *Nucleic Acids Res*. **35**, W375-W383 (2007).

14 Liu J, Yue Y, Han D et al. A METTL3-METTL14 complex mediates mammalian nuclear RNA N-6-adenosine methylation. *Nat Chem Biol*. **10**(2), 93-95(2014).

15 Huang J, Dong X, Gong Z et al. Solution structure of the RNA recognition domain of METTL3-METTL14 N(6)-methyladenosine methyltransferase. *Protein Cell*. **10**(4), 272-284 (2018).

16 Hsiao K, Zegzouti H, Goueli SA. Methyltransferase-Glo: a universal, bioluminescent and homogenous assay for monitoring all classes of methyltransferases. *Epigenomics*. **8**, 321-339 (2016).

17 Chen Z-L, Meng J-M, Cao Y et al. A high-speed search engine pLink 2 with systematic evaluation for proteome-scale identification of cross-linked peptides. *Nature Commun*. **10**(1), 3404 (2019).

18 Graham M, Combe C, Kolbowski L, Rappsilber J. xiView: A common platform for the downstream analysis of Crosslinking Mass Spectrometry data. *bioRxiv*.

DOI: https://doi.org/10.1101/561829

19 Czaplewski C et al. Recent developments in data-assisted modeling of flexible proteins. *Front Mol Biosci*. **8**, 765562 (2021).

20 Wang J-H, Gong Z, Dong X et al. Preferential cross-linking of the stereospecific complex over the encounter complexes by DOPA2, a faster cross-linker than DSS. *bioRxiv*.

DOI: https://doi.org/10.1101/2022.06.06.494913

21 Gong Z, Ding Y-H, Dong X et al. visualizing the ensemble structures of protein complexes using chemical cross-linking coupled with mass spectrometry. *Biophys Rep*. **1**, 127-138 (2015).

22 Wang X, Feng J, Xue Y et al. Structural basis of N(6)-adenosine methylation by the METTL3-METTL14 complex. *Nature*. **534**, 575-578 (2016).

23 Tunyasuvunakool K, Adler J, Wu Z et al. Highly accurate protein structure prediction for the human proteome. *Nature*. **596**, 590-596 (2021).

24 Bermejo GA, Clore GM, Schwieters CD. Smooth statistical torsion angle potential derived from a large conformational database via adaptive kernel density estimation improves the quality of NMR protein structures. *Protein Sci*. **21**, 1824-1836 (2012).

25 Schwieters CD, Clore GM. A pseudopotential for improving the packing of ellipsoidal protein structures determined from NMR data. *J Phys Chem B*. **112**, 6070-6073 (2008).
